# Supplementary material for: Pyruvate Dehydrogenase Kinase 1 inhibition mediated oxidative phosphorylation enhancement in cartilage promotes osteoarthritis progression
Source: BMC Musculoskelet Disord. 2023 Jul 20;24:597. doi: 10.1186/s12891-023-06585-6 (PMC10357736; doi:10.1186/s12891-023-06585-6)
Supplement: Supplementary file 1 — Additional file 1. [file 12891_2023_6585_MOESM1_ESM.zip › BP.Gsea.1653623667859/GOBP_RETROGRADE_VESICLE_MEDIATED_TRANSPORT_GOLGI_TO_ENDOPLASMIC_RETICULUM.html]

Details for gene set GOBP\_RETROGRADE\_VESICLE\_MEDIATED\_TRANSPORT\_GOLGI\_TO\_ENDOPLASMIC\_RETICULUM[GSEA]

|  || Dataset | 55457\_collapsed\_to\_symbols.cls.cls #OA\_versus\_normal.cls.cls #OA\_versus\_normal\_repos |
| Phenotype | cls.cls#OA\_versus\_normal\_repos |
| Upregulated in class | OA |
| GeneSet | GOBP\_RETROGRADE\_VESICLE\_MEDIATED\_TRANSPORT\_GOLGI\_TO\_ENDOPLASMIC\_RETICULUM |
| Enrichment Score (ES) | 0.5277065 |
| Normalized Enrichment Score (NES) | 2.1038482 |
| Nominal p-value | 0.0 |
| FDR q-value | 0.0063099787 |
| FWER p-Value | 0.118 |
Table: GSEA Results Summary

  

Fig 1: Enrichment plot: GOBP\_RETROGRADE\_VESICLE\_MEDIATED\_TRANSPORT\_GOLGI\_TO\_ENDOPLASMIC\_RETICULUM      
 Profile of the Running ES Score & Positions of GeneSet Members on the Rank Ordered List

  

| SYMBOL | TITLE | RANK IN GENE LIST | RANK METRIC SCORE | RUNNING ES | CORE ENRICHMENT || 1 | COPE | COPI coat complex subunit epsilon [Source:HGNC Symbol;Acc:HGNC:2234] | 15 | 1.136 | 0.0629 | Yes |
| 2 | COPZ2 | COPI coat complex subunit zeta 2 [Source:HGNC Symbol;Acc:HGNC:19356] | 19 | 1.109 | 0.1252 | Yes |
| 3 | KDELR3 | KDEL endoplasmic reticulum protein retention receptor 3 [Source:HGNC Symbol;Acc:HGNC:6306] | 77 | 0.950 | 0.1744 | Yes |
| 4 | ERGIC3 | ERGIC and golgi 3 [Source:HGNC Symbol;Acc:HGNC:15927] | 299 | 0.751 | 0.1995 | Yes |
| 5 | COPZ1 | COPI coat complex subunit zeta 1 [Source:HGNC Symbol;Acc:HGNC:2243] | 421 | 0.697 | 0.2294 | Yes |
| 6 | LMAN2 | "lectin, mannose binding 2 [Source:HGNC Symbol;Acc:HGNC:16986]" | 485 | 0.666 | 0.2620 | Yes |
| 7 | COPB2 | COPI coat complex subunit beta 2 [Source:HGNC Symbol;Acc:HGNC:2232] | 504 | 0.658 | 0.2978 | Yes |
| 8 | GOLPH3L | golgi phosphoprotein 3 like [Source:HGNC Symbol;Acc:HGNC:24882] | 521 | 0.652 | 0.3333 | Yes |
| 9 | RER1 | retention in endoplasmic reticulum sorting receptor 1 [Source:HGNC Symbol;Acc:HGNC:30309] | 724 | 0.588 | 0.3508 | Yes |
| 10 | BNIP1 | BCL2 interacting protein 1 [Source:HGNC Symbol;Acc:HGNC:1082] | 824 | 0.563 | 0.3748 | Yes |
| 11 | COG4 | component of oligomeric golgi complex 4 [Source:HGNC Symbol;Acc:HGNC:18620] | 966 | 0.525 | 0.3935 | Yes |
| 12 | DNAJC28 | DnaJ heat shock protein family (Hsp40) member C28 [Source:HGNC Symbol;Acc:HGNC:1297] | 1093 | 0.498 | 0.4118 | Yes |
| 13 | NBAS | NBAS subunit of NRZ tethering complex [Source:HGNC Symbol;Acc:HGNC:15625] | 1132 | 0.490 | 0.4364 | Yes |
| 14 | KDELR2 | KDEL endoplasmic reticulum protein retention receptor 2 [Source:HGNC Symbol;Acc:HGNC:6305] | 1486 | 0.431 | 0.4333 | Yes |
| 15 | ARF3 | ADP ribosylation factor 3 [Source:HGNC Symbol;Acc:HGNC:654] | 1572 | 0.418 | 0.4502 | Yes |
| 16 | RAB6A | "RAB6A, member RAS oncogene family [Source:HGNC Symbol;Acc:HGNC:9786]" | 1599 | 0.413 | 0.4715 | Yes |
| 17 | COG7 | component of oligomeric golgi complex 7 [Source:HGNC Symbol;Acc:HGNC:18622] | 1691 | 0.400 | 0.4870 | Yes |
| 18 | USE1 | unconventional SNARE in the ER 1 [Source:HGNC Symbol;Acc:HGNC:30882] | 1723 | 0.395 | 0.5069 | Yes |
| 19 | COPA | COPI coat complex subunit alpha [Source:HGNC Symbol;Acc:HGNC:2230] | 1959 | 0.366 | 0.5092 | Yes |
| 20 | ARF5 | ADP ribosylation factor 5 [Source:HGNC Symbol;Acc:HGNC:658] | 2027 | 0.358 | 0.5242 | Yes |
| 21 | KDELR1 | KDEL endoplasmic reticulum protein retention receptor 1 [Source:HGNC Symbol;Acc:HGNC:6304] | 2227 | 0.336 | 0.5277 | Yes |
| 22 | TMEM115 | transmembrane protein 115 [Source:HGNC Symbol;Acc:HGNC:30055] | 2735 | 0.276 | 0.5039 | No |
| 23 | SCFD1 | sec1 family domain containing 1 [Source:HGNC Symbol;Acc:HGNC:20726] | 2935 | 0.257 | 0.5029 | No |
| 24 | ATP9A | ATPase phospholipid transporting 9A (putative) [Source:HGNC Symbol;Acc:HGNC:13540] | 3398 | 0.212 | 0.4789 | No |
| 25 | RAB33B | "RAB33B, member RAS oncogene family [Source:HGNC Symbol;Acc:HGNC:16075]" | 3673 | 0.188 | 0.4682 | No |
| 26 | ERGIC2 | ERGIC and golgi 2 [Source:HGNC Symbol;Acc:HGNC:30208] | 4126 | 0.144 | 0.4411 | No |
| 27 | GOLPH3 | golgi phosphoprotein 3 [Source:HGNC Symbol;Acc:HGNC:15452] | 4420 | 0.119 | 0.4250 | No |
| 28 | TAPBP | TAP binding protein [Source:HGNC Symbol;Acc:HGNC:11566] | 4527 | 0.111 | 0.4231 | No |
| 29 | ATP9B | ATPase phospholipid transporting 9B (putative) [Source:HGNC Symbol;Acc:HGNC:13541] | 4606 | 0.104 | 0.4229 | No |
| 30 | STX18 | syntaxin 18 [Source:HGNC Symbol;Acc:HGNC:15942] | 4968 | 0.075 | 0.3990 | No |
| 31 | RINT1 | RAD50 interactor 1 [Source:HGNC Symbol;Acc:HGNC:21876] | 5863 | 0.004 | 0.3297 | No |
| 32 | ARF4 | ADP ribosylation factor 4 [Source:HGNC Symbol;Acc:HGNC:655] | 6894 | -0.072 | 0.2536 | No |
| 33 | HTT | huntingtin [Source:HGNC Symbol;Acc:HGNC:4851] | 7641 | -0.130 | 0.2029 | No |
| 34 | ARCN1 | archain 1 [Source:HGNC Symbol;Acc:HGNC:649] | 8112 | -0.164 | 0.1756 | No |
| 35 | BICD2 | BICD cargo adaptor 2 [Source:HGNC Symbol;Acc:HGNC:17208] | 8253 | -0.175 | 0.1746 | No |
| 36 | RAB6B | "RAB6B, member RAS oncogene family [Source:HGNC Symbol;Acc:HGNC:14902]" | 8309 | -0.179 | 0.1804 | No |
| 37 | GBF1 | golgi brefeldin A resistant guanine nucleotide exchange factor 1 [Source:HGNC Symbol;Acc:HGNC:4181] | 8461 | -0.191 | 0.1794 | No |
| 38 | UVRAG | UV radiation resistance associated [Source:HGNC Symbol;Acc:HGNC:12640] | 8555 | -0.198 | 0.1834 | No |
| 39 | SEC22B | "SEC22 homolog B, vesicle trafficking protein [Source:HGNC Symbol;Acc:HGNC:10700]" | 8614 | -0.202 | 0.1902 | No |
| 40 | PLPP3 | phospholipid phosphatase 3 [Source:HGNC Symbol;Acc:HGNC:9229] | 9906 | -0.312 | 0.1074 | No |
| 41 | KIF1C | kinesin family member 1C [Source:HGNC Symbol;Acc:HGNC:6317] | 9942 | -0.316 | 0.1225 | No |
| 42 | TMED10 | transmembrane p24 trafficking protein 10 [Source:HGNC Symbol;Acc:HGNC:16998] | 9993 | -0.320 | 0.1367 | No |
| 43 | PICK1 | protein interacting with PRKCA 1 [Source:HGNC Symbol;Acc:HGNC:9394] | 11234 | -0.461 | 0.0663 | No |
| 44 | PITPNB | phosphatidylinositol transfer protein beta [Source:HGNC Symbol;Acc:HGNC:9002] | 11527 | -0.505 | 0.0720 | No |
| 45 | BET1L | Bet1 golgi vesicular membrane trafficking protein like [Source:HGNC Symbol;Acc:HGNC:19348] | 12084 | -0.615 | 0.0635 | No |
Table: GSEA details [plain text format]

  

Fig 2: GOBP\_RETROGRADE\_VESICLE\_MEDIATED\_TRANSPORT\_GOLGI\_TO\_ENDOPLASMIC\_RETICULUM      
 Blue-Pink O' Gram in the Space of the Analyzed GeneSet

  

Fig 3: GOBP\_RETROGRADE\_VESICLE\_MEDIATED\_TRANSPORT\_GOLGI\_TO\_ENDOPLASMIC\_RETICULUM: Random ES distribution      
 Gene set null distribution of ES for **GOBP\_RETROGRADE\_VESICLE\_MEDIATED\_TRANSPORT\_GOLGI\_TO\_ENDOPLASMIC\_RETICULUM**
